# DHEA supplementation improves endometrial HOXA-10 mRNA expression in poor responders

**DOI:** 10.4274/jtgga.2017.0054

**Published:** 2017-12-15

**Authors:** Önder Çelik, Mustafa Acet, Aytaç İmren, Nilüfer Çelik, Aynur Erşahin, Lebriz Hale Aktun, Barış Otlu, Sudenaz Çelik, Eray Çalışkan, Cihat Ünlü

**Affiliations:** 1 Private Clinic, Obstetrics and Gynecology, Uşak, Turkey; 2 Department of Obstetrics and Gynecology, Medipol University Faculty of Medicine, İstanbul, Turkey; 3 Clinic of Obstetric and Gynecology, Medical Park Hospital, Uşak, Turkey; 4 Clinic of Biochemistry, Behçet Uz Children’s Hospital, İzmir, Turkey; 5 Department of Obstetrics and Gynecology, Bahçeşehir University Faculty of Medicine, İstanbul, Turkey; 6 Department of Medical Microbiology, İnönü University Faculty of Medicine, Malatya, Turkey; 7 Kent College Güzelbahçe High School, İzmir, Turkey; 8 Department of Obstetrics and Gynecology, Bahçeşehir University Faculty of Medicine, Kocaeli, Turkey; 9 Department of Obstetrics and Gynecology, Acıbadem University Faculty of Medicine, İstanbul, Turkey

**Keywords:** Endometrium, DHEA, homeobox genes, poor responder

## Abstract

**Objective:**

The study was planned to investigate whether DHEA supplementation had an impact on endometrial receptivity in women who were poor responders (POR).

**Material and Methods:**

Twenty-eight POR women who were undergoing hysteroscopy and five fertile control subjects were included. The POR women were equally subdivided into two separate groups as patients who were currently using DHEA and those who were not. Endometrial samples of the subjects were obtained during hysteroscopy at the late follicular phase. Expression levels of endometrial HOXA-10, HOXA-11, and LIF mRNA were measured with the using real-time polymerase chain reaction. Spontaneous clinical pregnancy rates were also noted.

**Results:**

Compared with POR women who were not given DHEA, upregulated endometrial HOXA-10 (7.33-fold) and HOXA-11 (2.39-fold) mRNA expression were detected in POR women on DHEA. The increase in HOXA-10 mRNA was significant (p<0.03). The fold increase in HOXA-11 mRNA was found as 2.39, which indicated a positive upregulation. However, this fold increment was insignificant (p<0.45). An insignificant increase in spontaneous clinical pregnancy rates in POR women on DHEA (53.3%) was observed compared with POR women who were not given DHEA (43.8%).

**Conclusion:**

Oral DHEA supplementation in POR upregulates endometrial HOXA-10 mRNA expression, which is known to positively modulate endometrial receptivity.

## INTRODUCTION

A fall in androgens, their metabolites, and DHEA decrease progressively during reproductive period (1,2,3). DHEA is a critical substrate for sex steroid production in aging women (4). A recent review reported that ovarian synthesis of DHEA decreases with age (5). A little more than half of DHEA is of adrenal origin, the remaining amount is released from the ovary (6). Similarly, the pool of androgens including DHEA decreases gradually with age (7,8). Local endometrial androgen levels also decrease due to a remarkable decline in the synthesis of adrenal androgens. All these could be responsible for the decline in reproductive performance in aging women (7).

The decrease in ovarian reserve is not only a reality in older women, it also might occur in young women with infertility (1,2). DHEA looks like a breakthrough therapeutic medication in improving ovarian responses in poor-responder (POR) patients. Despite the common use of DHEA as a supplement in POR, the exact mechanism of DHEA action on reproductive events remains speculative. To date, information related to the impact of DHEA on reproductive outcome has largely focused on the potential for the count of retrieved oocytes. Concordantly, androgens have an important role in the early phase of follicular growth, before follicles become follicle-stimulating hormone (FSH) sensitive. They inhibit apoptosis in follicles and improve the action of FSH. In line with these findings, it has been reported that the use of androgen patches increased both pregnancy and take home baby rates in POR women (9,10).

As apart from the decreased retrieval of mature oocytes, the decline in endometrial receptivity might adversely affect reproductive performance in aging women. Accordingly, the reality of endometrial aging and the critical role of androgens have been noted (11). Such as with ovarian aging, it is reasonable to assume a decline in efficiency of endometrium receptivity during the advancing reproductive period. It is most likely that androgenic endometrial milieu changes as women age because DHEA levels significantly decrease with advancing age (1,2).

Some extragonadal tissues consist of steroidogenic enzymes that may convert DHEA to active androgens and estrogens. This cell-specific intracrinology may cause the local production of potent metabolites in accordance with cell-specific requisitions. In support, a regulatory effect of androgens on endometrial cell survival has been reported (12). In view of the above mentioned facts, we hypothesized that DHEA supplementation in POR women may improve reproductive performance through other mechanisms than the number of retrieved oocytes. The endometrium, therefore, may be the most likely area where potential positive effects of DHEA are seen. Although expression of androgen receptor (AR) has been reported in the human endometrium (13), the effect of DHEA on endometrium receptivity genes remained elusive. There is no controlled study investigating the possible effect of DHEA supplementation on homeobox genes (HOXA-10 and HOXA-11) and leukemia-inhibitory factor (LIF). These are the key receptivity genes that regulate decidualization and implantation rates (14,15). To detect the possible influence of DHEA on receptivity we compared the expression intensities of endometrial HOXA-10, HOXA-11, and LIF mRNA in POR women who were given DHEA and others that were not.

## MATERIAL AND METHODS

The primary outcome of this work was to investigate the hypothesis that DHEA improves endometrium receptivity in POR. The secondary outcome was to determine spontaneous clinical pregnancy rates. The presence of at least two of the following three features were accepted as a POR (16): (i) Maternal age ≥40 years; (ii) Previous history of retrieving fewer than 3 oocytes; (iii) Previous history of decreased ovarian reserve [antral follicle count (AFC), 5-7 follicles or anti-müllerian hormone (AMH), 0.5-1.1 ng/mL]. Some women in our study had lower levels of AMH than 0.5 ng/mL. Most of the POR patients in the study group were aged less than 40 years and met the criteria in sections (ii) and (iii).

Twenty-eight women with POR fulfilled the eligibility criteria and participated in the study. At the initial visit, although the participants had a diagnosis of POR, they underwent confirmatory ultrasound examination for their AFC. Blood was also obtained from all participants for AMH measurement. Following verbal informed consent and local Institutional Review Board approval, the POR women were equally divided in to two groups as subjects who were currently using DHEA (n=14) and those were not (n=14). POR women with a history of taking DHEA for at least 6 weeks were included in the DHEA treatment group. This time interval was chosen because of the early follicular growth induced by DHEA that occurs within 2 months of treatment. The POR women who were not using DHEA were accepted as the control group. Five fertile subjects were accepted as the second control group. POR women in the treatment group received oral DHEA with 25 mg/TID. Women with untreated hydrosalpinges, submucous or intramural leiomyomas, endometrial polyps, male factor infertility, and tubal factor infertility were excluded. Women with endocrine disorders such as insulin-dependent diabetes mellitus, congenital adrenal hyperplasia, thyroid diseases, and hyperprolactinemia were also excluded. Women with a history of allergy to DHEA were not included. Endometrial thickness was measured at the late follicular phase following hysteroscopy and serum samples were obtained for hormonal evaluation.

In order to establish endometrial causes of former failed in vitro fertilization (IVF) cycles and local endometrial damage, the decision for hysteroscopy was taken for both groups of POR participants. All subjects underwent hysteroscopy at the late follicular phases and endometrial samples were obtained. Following repetitive washing of samples with a saline solution, they were transferred into an RNA stabilization buffer and stored at -80 °C. POR subjects were left for a 4-5 month waiting period after hysteroscopy; if the women did not achieve pregnancy during this time period they underwent in vitro fertilization/intracytoplasmic sperm injection (IVF/ICSI).

### Real-time polymerase chain reaction (RT-PCR)

### Sample preparation and RNA isolation

Both the RT-PCR method used for measuring expression levels of endometrial mRNA and comparative RNA expression analysis methods were the same as used in the recent study conducted by our team; more information can be found elsewhere (17). Unless otherwise specified, the kits used in this study were obtained from Qiagen, Hilden, Germany.

### Reverse transcription cDNA synthesis

Complementary DNA was prepared by using a QuantiTect Reverse Transcription kit (17).

### RT-PCR analysis of homeobox and LIF genes

Both positive controls and other genes were prepared using PrimerDesign to analyze the efficacy of the PCR reaction. The mRNA levels of sampling tissues were normalized to that of the house-keeping gene (β-actin) mRNA level. RT-PCR results are expressed as cycle threshold (Ct), delta Ct (ΔCt) and ddCt (ΔΔCt). For the calculation of average Ct values, each endometrial sample was studied three times. The sequence and accession numbers of all primers designed to be used as forward and reverse primers for RT-PCR were: HOXA-10(NM_018951),F-5’-GGTTTGTTCTGACTTTTTGTTTCT-3’, R-5’TGACACTTAGGACAATATCTATCTCTA-3’;HOXA-11(NM_005523),F-5’-AGTTCTTTCTTCAGCGTCTACATT-3’, R-5’TTTTTCCTTCATTCTCCTGTTCTG-3’; LIF(NM_002309),F-5’GGAGGTCACTTGGCATTCAG-3’, R-5’GGAAGAGAACGAAGAACCTACC-3’; and ACTB(NM_001101), F-5’GCAAGCAGGAGTATGACGAGT-3’, R-5’CAAGAAAGGGTGTAACGCAACTAA-3’.

### Statistical analysis

The expression of the studied genes was determined using the 2-ΔΔCT method. All data were normalized according to the ACTB gene (β-actin) mRNA content. The normalized gene expression of POR women was divided by the normalized gene expression of the control subjects. The fold increase were considered positive or an up-regulation for transcript overexpression when the corresponding mRNA level was at least 2-fold higher than that of the initial transcript expression, negative or down-regulation, if lower than 2-fold. The Kolmogorov-Smirnov test showed normal distributions of data. The ANOVA test with post hoc Tukey’s procedure and Mann-Whitney U test were used for analyzing continuous variables. Pearson’s Chi-square test was used for analyzing other data. P<0.05 was accepted as statistically significant. The results are given as mean and standard deviation (SD).

## RESULTS

POR women on DHEA and without DHEA had similar age, AFC, FSH, and endometrial thickness (Table 1). Significantly lower AMH levels were detected in women on DHEA compared with women who were not taking DHEA (0.33±0.23 vs. 0.64±0.12 μg/mL). The mean age of the fertile controls was 33.1+2.3 years. The mean age of the POR women on DHEA (33.0±4.57 years), those without DHEA (33.2±6.18 years), and controls were similar. The mean baseline FSH (5.6±0.5 mIU/mL) levels of the fertile group were lower than in POR women on DHEA (10.8±1.12 mIU/mL). The mean baseline endometrial thickness of the fertile group (7.4±0.5) and women on DHEA (7.73±0.79) were similar. The mean baseline AFCs of the fertile group (4.67±1.87) was higher than in POR women on DHEA (1.80±0.67). Trends toward an increase in clinical spontaneous pregnancy rates in POR women on DHEA were detected compared with the POR women who were not given DHEA (53.3% vs. 43.8%). However, the difference was found insignificant (p<0.59). One subject had early pregnancy loss among the POR women on DHEA, and 3 of the 14 women who were not given DHEA had pregnancy loss (Table 1).

**Figure 1:**
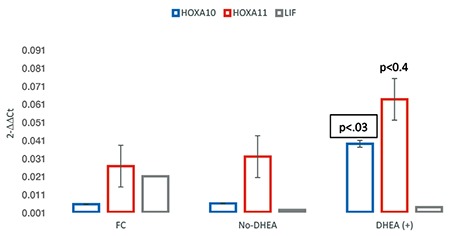
The relative gene expression was determined using the 2^−ΔΔCT^ method. All data were compared with the fertile control group and normalized to ACTB gene (actin, beta) mRNA content. No-DHEA; POR women who were not given DHEA, DHEA (+); POR women on DHEA FC; fertile control; POR: poor responders

The expression levels of endometrial HOXA-10 and HOXA-11 mRNA of subjects who were not given DHEA and fertile women were the same (p<0.44 and p<0.25 respectively). Expression levels of LIF mRNA were found to be lower in the endometrium of POR subjects who were not given DHEA as compared with the fertile controls. However, the difference was noted as insignificant (p<0.48). Upregulated HOXA-10 and 11 mRNA expressions were found in women taking DHEA. POR women on DHEA showed a 7.33-fold increment in HOXA-10 mRNA expression, and a 2.39-fold increment was detected in HOXA-11 mRNA expression. Only the increment in HOXA-10 mRNA expression was significant (p<0.03). The fold increment in HOXA-11 mRNA after DHEA supplementation was found as 2.39, which was greater than two, thus indicating an upregulation. However, this increment was not significant (p<0.45). Likewise, an insignificant increase in LIF mRNA expression (1.76-fold, p<0.36) was detected after DHEA supplementation. Due to the fold rise in LIF mRNA following DHEA being smaller than 2-fold, it was considered to be down-regulation (Table 2, Figure 1).

## DISCUSSION

The two main results arising from this study are the increased endometrial HOXA-10 mRNA expression and the spontaneous pregnancy rates being higher than those reported in the literature. Although DHEA supplementation increases follicle recruitment, potentializes gonadotrophin effect, reduces follicle apoptosis, and enhances IGF-1 levels, how DHEA improves fertility outcomes is still not accurately known (18,19,20). Barad et al. (21) reported the clinical pregnancy rates in DOR women on DHEA as 10.9-28.1%, and half of the pregnancies occurred spontaneously. In the current study, more than 50% of POR women on DHEA (53.3%) conceived within 4-5 months after the hysteroscopy. In line with our results, a study conducted by Fusi et al. (22) showed that DHEA improved the chance of spontaneous pregnancies in POR women. High spontaneous pregnancy rates in POR patients on DHEA might be due to the positive impact of DHEA on the endometrial microenvironment, as well as the positive impact on oocyte development.

In order to clarify enhanced endometrial receptivity following DHEA administration, both average 2-ΔCt and fold change values for each gene were measured. The main result from the current study is the 7.3-fold rise in HOXA-10 and 2.3-fold rise in HOXA-11 mRNA expression after DHEA. Based on upregulated expressions of both genes, we can strongly suggest that exogenous DHEA improves endometrial receptivity. However, it is not evident whether DHEA has a direct effect on endometrial receptivity or its function only as a precursor to estrogens. Accordingly, androgens are not solely a substrate for estrogen production but may also modulate the effects of estrogen in the endometrium (23). If we accept the idea that DHEA increases endometrial receptivity by transforming into estrogen, it is logical to believe that administration of exogenous estrogens should improve endometrial receptivity. Confirmation of our hypothesis comes from an egg donation study of women with advanced reproductive age (24). Estrogen priming of these women improves both endometrial thickness and implantation. However, increased implantation is not only due to the positive impact of estrogen. It should be remembered that good quality oocytes from healthy donors may overcome any age-related receptivity defect.

Peripheral interconversion of DHEA to active androgens, estrogens, and progesterone may be the first reason of the increased endometrial HOXA10 mRNA expression. It is a well-known fact that expression of homeobox genes are modulated by sex steroids (25). Concordantly, endometrial HOXA10 mRNA was found to be associated with circulating 17-β estradiol (25). Likewise, androgens are also regulators of the HOXA10 gene (26). As DHEA turns estrogens in peripheral tissues (27,28), exogenous DHEA can exhibit a positive impact on endometrial HOXA10 mRNA expression in POR women on DHEA.

The second possibility of the positive effect of DHEA on HOXA10 mRNA might be the substitution of androgens or estrogens because circulating levels of androgen and estrogen decrease with advancing age (29). Nevertheless, local transformation of DHEA of other steroids in the endometrium is restricted by the altered levels of steroidogenic enzymes (11). Some authors believe in the local production of estradiol in the endometrium, whereas others do not support this notion (30,31). The lack of aromatase activity and the existence of endometrial atrophy in postmenopausal women support the idea that the endometrium does not have the ability to produce local estrogen (11,30). In contrast, Bukulmez et al. (32) reported that mRNA expression of aromatase enzyme in cultured endometrial cells were up-regulated by androstenedione (33). If a rise in HOXA10 mRNA following DHEA is secondary to conversion of DHEA to estrogens, why does endometrial thickness not alter significantly? In contrast to the stimulatory effect of estrogen on the endometrium, DHEA does not exert a stimulatory impact on endometrium (7). In good agreement with this, Labrie et al. (34) reported that the stimulatory impact of DHEA on the vagina was not detected in the endometrium of postmenopausal women and their endometrium remained atrophic after one year of DHEA supplementation. Likewise, endometrial thickness of women on DHEA and those that were not given DHEA were similar in our study. Together, upregulation of endometrial HOXA-10 mRNA after DHEA treatment could be attributed to either DHEA itself or a function of its active compounds. If absolute androgen deficiency has a negative impact on endometrial androgen levels, we may suggest that exogenous DHEA may lead to a rise in local androgens that induce endometrial HOXA-10 mRNA expression.

AR have been shown in both pre- and postmenopausal human endometrium (35,36). Estrogen increases AR, and progesterone inhibits it (37,38). Therefore, a third possibility of augmented HOXA-10 expression after DHEA might be related to an AR enhancer effect of DHEA. The greatest support for our hypothesis come from the study by Qin et al. (39) who investigated the impact of DHEA on decidual PRL-related protein (dPRP), AR, and HOXA-10 expressions in mouse endometrial cells. They reported that DHEA had an insignificant effect on endometrial dPRP expression. The authors also noted that when given the dose of 100 nM, DHEA caused a significant increase in HOXA-10 mRNA. Moreover, they showed that DHEA-mediated upregulation in HOXA-10 was diminished by treatment with an AR antagonist. Together, if physiologic androgen deficiency in aging women leads to a decline in AR, we can suggest that exogenous DHEA can increase endometrial AR, which might lead to a rise in HOXA-10 (39).

The fourth possibility of improved receptivity after DHEA may be related to decidualization. By stimulating decidual prolactin production, androgens regulate decidualization (40,41). Exogenous DHEA might upregulate HOXA-10 mRNA expression because homeobox genes induce decidualization and pinopode formation (14,42).

There are some limitations to the current study. The study population is small in the studied groups. Alterations in mRNA expression are not confirmed by protein analyses. As opposed to our findings, patients with hyperandrogenism secondary to Polycystic Ovarian syndrome (PCOS) demonstrated low HOXA-10 and β3-integrin expression (43,44), suggesting androgens may have a detrimental impact on the endometrium. For this reason, one may suggest that the use of exogenous DHEA could impair the endometrial micro-milieu and that there is no need for androgen supplementation in POR women. Actually, DHEA may cause a PCO-like appearance in ovaries of POR women (45,46). However, as exogenous DHEA does not exactly mimic clinical and biochemical features of genuine PCOS, results obtained from subjects with PCO-like ovaries cannot be applied to POR women on DHEA.

**Figure 2:**
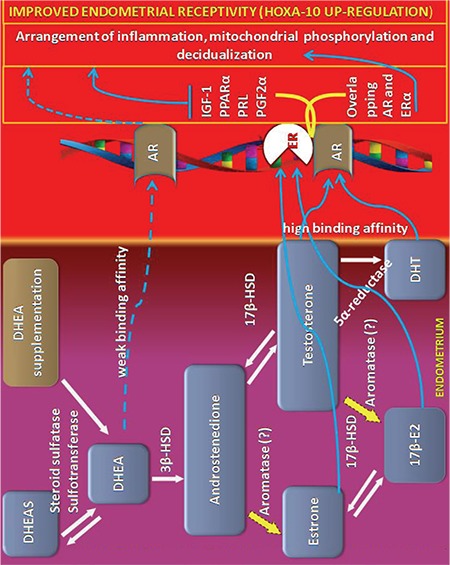
Abbreviated pathways to illustrate the possible mechanism of action of DHEA on endometrium receptivity.

Finally, we demonstrated for the first time that oral DHEA supplementation augments endometrial HOXA-10 mRNA expression. As well as the known possible positive effect on the count of oocytes retrieved, DHEA may increase implantation and pregnancy rates by modulating receptivity genes (17) or signal molecules (47). The receptivity enhancing effects of DHEA might be realized via transformation of DHEA to active metabolites (Figure 2). If DHEA indeed has a positive impact on endometrial receptivity, it can be used to enhance implantation rates in women with POR. Whatever the mechanism, the present study showed that DHEA exerted a positive effect on endometrial receptivity. If our results are supported by extensive studies, augmentation of endometrial receptivity with DHEA might be a key factor for the management of women with implantation failure.

## Figures and Tables

**Table 1:**
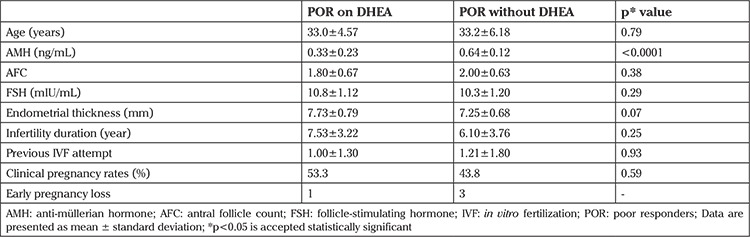
Clinical characteristics of POR women on DHEA and without DHEA

**Table 2:**
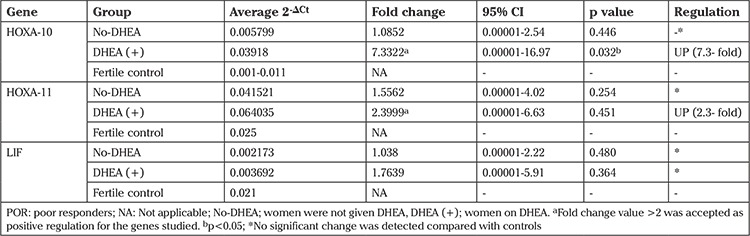
Comparison of expression levels of endometrial HOXA-10, HOXA-11, and LIF mRNA in the fertile control, POR women on DHEA, and without DHEA
